# Rodin: a streamlined metabolomics data analysis and visualization tool

**DOI:** 10.1093/bioadv/vbaf088

**Published:** 2025-04-11

**Authors:** Boris Minasenko, Dongxue Wang, Piera Cirillo, Nickilou Krigbaum, Barbara Cohn, Dean P Jones, Jeffrey M Collins, Xin Hu

**Affiliations:** Gangarosa Department of Environmental Health, Rollins School of Public Health, Emory University, Atlanta, GA, 30322, United States; Gangarosa Department of Environmental Health, Rollins School of Public Health, Emory University, Atlanta, GA, 30322, United States; Child Health and Development Studies, Public Health Institute, Berkeley, CA, 94709, United States; Child Health and Development Studies, Public Health Institute, Berkeley, CA, 94709, United States; Child Health and Development Studies, Public Health Institute, Berkeley, CA, 94709, United States; Division of Pulmonary, Allergy, Critical Care, and Sleep Medicine, Department of Medicine, School of Medicine, Emory University, Atlanta, GA, 30322, United States; Department of Infectious Disease, School of Medicine, Emory University, Atlanta, GA, 30322, United States; Gangarosa Department of Environmental Health, Rollins School of Public Health, Emory University, Atlanta, GA, 30322, United States; Division of Pulmonary, Allergy, Critical Care, and Sleep Medicine, Department of Medicine, School of Medicine, Emory University, Atlanta, GA, 30322, United States

## Abstract

**Summary:**

Recent advances in high-resolution mass spectrometry have revolutionized metabolomics, enabling the profiling of hundreds of thousands of metabolic features in a single experiment, with widespread applications across health sciences. To streamline analysis of metabolomics data, we developed Rodin, a Python-based application offering fast, efficient processing of large datasets via a web interface or programming library. Rodin integrates multiple stages of analysis, including feature preprocessing, statistical testing, interactive visualizations, and pathway analysis, generating outputs while tracking user-defined parameters within a single page. By enhancing the accessibility of tools for metabolomics data analysis, Rodin not only streamlines the workflow but also enhances analytic throughput by enabling a broader range of users to perform these analyses. Compared to other tools, Rodin excels in user-friendliness, ease of access, and seamless integration of multiple functionalities, enabling reproducible, efficient workflows for users of all computational skill levels.

**Availability and implementation:**

Web interface—https://rodin-meta.com/. Python library—https://github.com/BM-Boris/rodin.

## 1 Introduction

Metabolomics is an emerging branch of omics aimed at studying metabolites in biological systems. This field has rapidly advanced over the past decades, driven by enhanced selectivity, sensitivity, and coverage of high-resolution mass spectrometry (HRMS) and become an integral field of systems biology. The number of publications has been actively growing in recent years, underscoring the value of metabolomics in improving disease diagnostics, biomarker discovery, and understanding of biological mechanisms (Johnson *et al.* 2016).

The growth of metabolomics is facilitated by several analytical advancements in chromatography and mass spectrometry, along with the accompanying increase in throughput and the reduction in cost. Recent technical breakthroughs in the availability of HRMS have revolutionized the MS-based detection of metabolites with unprecedented mass resolution and accuracy (Johnson *et al.* 2016). Liquid chromatography-mass spectrometry (LC-MS) is widely applied due to its high sensitivity, reproducibility and versatility in measuring the masses of molecules and their adducts to determine their identity. Untargeted metabolomics, advantageous for its extensive and unbiased coverage of >100 metabolic pathways, usually yields over 50 000 metabolites for each study sample, with tens of millions of data points for medium-sized studies. The workflow of untargeted metabolomics spans data acquisition, pre-processing to correct for noise and variability, advanced statistical analysis, often followed by metabolic pathway analysis that transforms unannotated features into biologically meaningful results. As these technologies become more accessible, there is a growing need for fast, efficient, and user-friendly tools for analyzing big data.

The computational workflow analyzing untargeted metabolomics data begins with mass spectrometry data extraction using common software tools such as apLCMS and XCMS, which construct extracted ion chromatograms and generate feature tables that contain mass-to-charge (*m*/*z*) and retention time as identifiers for each metabolic feature, along with the feature’s peak intensity for the samples (Smith *et al.* 2006, Yu *et al.* 2009). Subsequent data analysis includes data preprocessing and statistical analysis to select features that are most relevant to the research question. Pathway analysis provides insight into biological processes and is often followed by further statistical analysis, including univariate, multivariate, and machine learning approaches, to validate and prioritize certain features or pathways.

We have developed Rodin (https://rodin-meta.com), a Python-based application with both web interface and Python programming options (https://github.com/BM-Boris/rodin), to streamline metabolic feature selection, visualization and pathway analysis. Built on powerful libraries like *NumPy* (Harris *et al.* 2020) and *Pandas* for large dataset processing, and *Statsmodels* (Seabold & Perktold 2010) with *Scikit-learn* (Pedregosa *et al.* 2011) for statistical analysis, Rodin excels in handling large datasets with superior speed and stability, compared to many R packages (Table 1). The intuitive web interface integrates steps from feature table preprocessing to pathway analysis on a single page, eliminating the need for multiple file transfers. Researchers can easily share the saved webpage (enabled by built-in feature of web browsers to export as html or PDF format) or notebooks (if using the Python library) to allow peer verification. Rodin also features interactive visualization built on *Plotly* so that users can easily select any metabolic feature or study group and customize layouts to visually identify patterns, clusters, and individual metabolite changes relevant to the research question.

**Table 1.** Usability comparison of Rodin to other software.

Software	Runtime	Visualization and interactivity	Capabilities and limitations
MetaboAnalyst 6.0 (web interface) (Pang *et al.* 2024)	>30 min	Interactive visualizations available, but users must navigate multiple interfaces to access the complete results	Supports up to 5000 features; does not allow extraction of data from intermediate pre-processing and analytic steps; requires extra processing to incorporate non-significant features into pathway analysis
Mummichog 2.0 (Li *et al.* 2013)	N/A^a^	Produces static outputs; manual linking of annotations to pathways is necessary	Focused solely on pathway analysis without integrated statistical testing for feature selection; limited options for annotation refinement
xmsPANDA (github)	>240 min	Generates several intermediate files that must be manually inspected, resulting in low efficiency	Installation requires managing numerous dependencies and specific R versions; performs feature selection without an integrated pathway analysis
XCMS online MS-DIAL (Tautenhahn *et al.* 2012)	N/A^a^	Limited visualization tools (designed mainly for data extraction)	Primarily designed for data extraction; offers minimal statistical analysis capabilities
Rodin	<2 min	Provides fully interactive, customizable real-time visualizations that display the complete dataset without switching between interfaces	Handles datasets with unlimited number of features; offers full dataset downloads and a fully integrated, end-to-end pipeline from feature selection to automated pathway analysis; uses only minimal and well-maintained dependencies to streamline the workflow

a Runtime metrics are not applicable, as the software only provides a partial workflow.

In summary, Rodin offers a fast, convenient, and interactive solution for large-scale metabolomics data analysis (Table 1). Originally designed for untargeted metabolomics, its flexible and intuitive interface also supports statistical analysis and visualization of other big data types such as transcriptomics or epigenomics. The streamlined, single-document design enhances the efficiency of delivering analysis results while ensuring reproducibility and rigor in alignment with the FAIR principles (Findable, Accessible, Interoperable and Reusable).

## 2 Features and implementation

### 2.1 Interactive design concept

Rodin uses Python as the core programming language for efficient computation and flexibility in implementing complex data analysis algorithms. The backend computational functions of Rodin are powered by a custom Python library available at https://github.com/BM-Boris/rodin. Integration between the web interface and the backend computational modules is achieved through Django's view functions, which handle user requests and coordinate the execution of computational tasks. Django was chosen for its robustness and seamless integration with Python's extensive ecosystem of scientific libraries. When a user interacts with the web interface, by uploading data or initiating an analysis, the algorithm invokes the appropriate functions from the custom library and returns the results to the user in real time. Rodin uses a relational database managed by Django's Object-Relational Mapping (ORM) system. This database stores user’s uploaded data, parameter selection, and results temporarily and securely during the analysis process. All user data is deleted upon session termination or after a predefined period of inactivity, in accordance with the application's privacy policy.

Rodin is hosted on the Amazon Web Services (AWS) infrastructure within Emory University's environment. The secure and access-controlled server environment is configured with adequate computational resources to handle the demands of metabolomic data processing. The application has been tested across various operating systems and web browsers, including mobile devices, to ensure compatibility and consistent user experience.

Upon data loading, Rodin checks the data for readiness for analysis, and enables functions consisting of feature preprocessing, statistical analysis, interactive visualization and pathway analysis. The interface is designed to be intuitive, user-friendly and streamlined to minimize the need for navigation between different sections. JavaScript is implemented for dynamic and immediate updates of each individual section without full page reloads. Detailed explanations of the expected data input, functionalities and tips on selection of parameters and statistical tests are provided in the guide page on the web interface (also included in Supplemental Material). Tutorials are available to help users familiarize themselves with the Python library’s capabilities and workflows.

In summary, Rodin integrates the Django framework and Python's scientific libraries to provide a robust, efficient, and user-friendly platform for metabolomic data analysis. It builds on well-established libraries while introducing novelty through the seamless integration of these techniques into a unified platform. Leveraging AWS infrastructure, Rodin ensures scalability and reliability, enabling large-scale data processing within a streamlined and uninterrupted workflow.

### 2.2 Workflow

**Figure 1.**
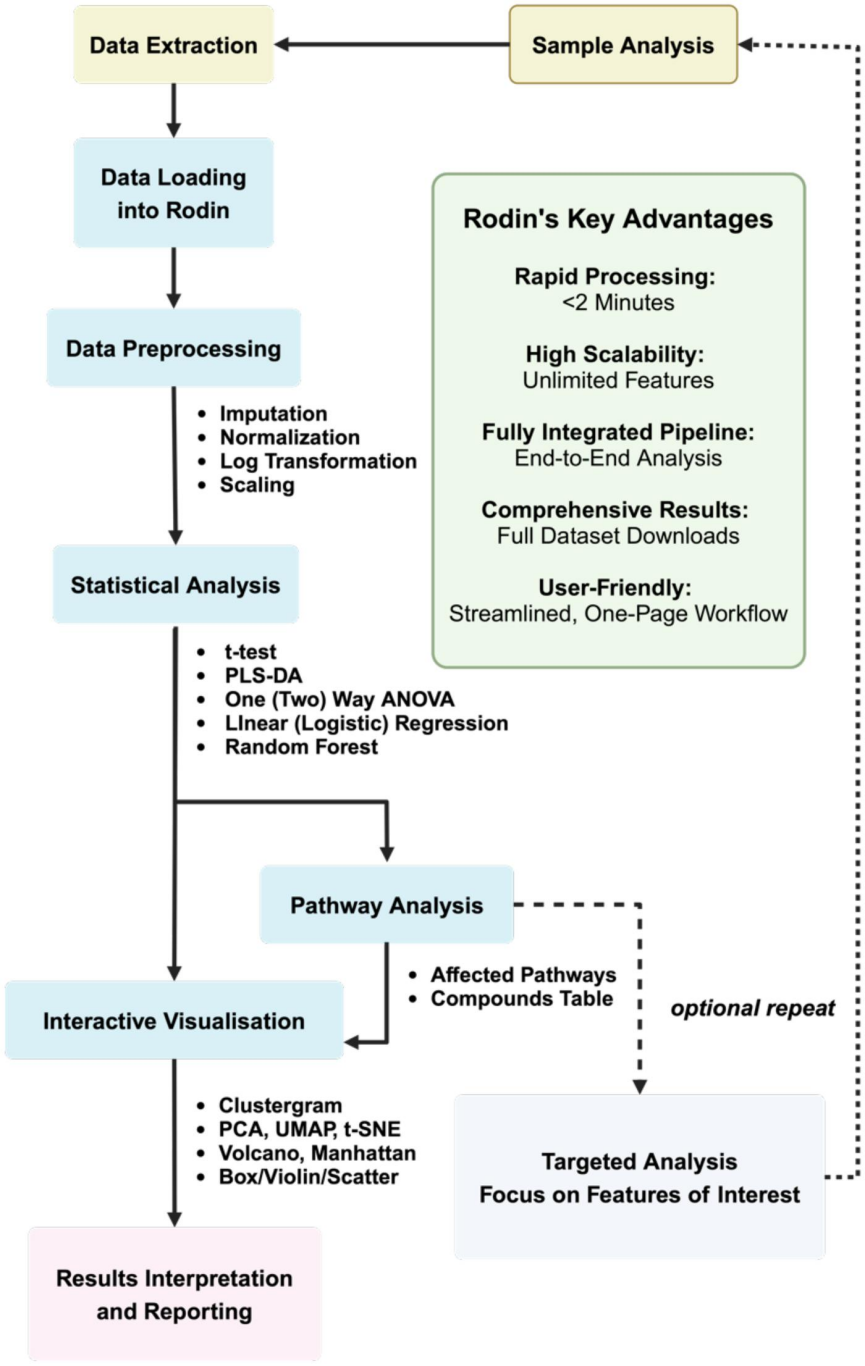
Workflow diagram illustrating the complete untargeted metabolomics data analysis and how Rodin facilitates integrated feature preprocessing and selection, statistical and pathway analyses, and data visualization. Created in BioRender. Hu, X. (2025) https://BioRender.com/589dxsv.

The workflow design of Rodin is illustrated in Fig. 1 and described in sections below.

#### 2.2.1 Data upload

The app accepts files in csv or txt format, each not exceeding 150 megabytes in size. For untargeted analyses, users should prepare a Feature Table where the first two columns contain the mass-to-charge ratios (m/z) and retention times of the detected features. The subsequent columns should correspond to identifiers of samples, with each cell containing the measured intensity of a feature in a particular sample. This format aligns with standard outputs from apLCMS (Yu *et al.* 2009), xMSanalyzer (Uppal *et al.* 2013), and XCMS (Smith *et al.* 2006) extraction. For targeted analyses, where specific metabolites have been annotated, the first column of the Feature Table should list unique metabolite annotations, with subsequent columns containing metabolite intensities or concentrations of study samples. In addition to the Feature Table, users must provide a Class Labels file with sample metadata. The first column contains sample IDs in the rows, matching exactly with those in the Feature Table, with subsequent columns including factors of interest such as treatment groups, doses, or time points.

Before proceeding, Rodin performs a series of validation checks to ensure data integrity. The application verifies that the sample IDs in both the Feature Table and the Class Labels files match and are in the same sequence. If discrepancies are detected—such as mismatched IDs or differences in the number of samples—Rodin alerts the user and specifies which sample IDs are absent or inconsistent.

#### 2.2.2 Data preprocessing

Rodin offers options to filter metabolic features that have high ratios of missing or zero values—a common occurrence in metabolomics with compound levels below detection limits. Remaining missing values are imputed with zeros. Alternative imputation strategies—such as those based on detection limits or half the minimum value—were not adopted, as they may not optimally capture the variability in untargeted metabolomics, particularly with Orbitrap mass spectrometry data. Users can opt to exclude samples with missing class labels. Based on the practical demands commonly seen in untargeted metabolomics, Rodin provides normalization options of quantile normalization, which adjusts data distributions across samples to be statistically similar, and total intensity scaling, which normalizes each sample so that total detected intensities for each sample are equal. Additionally, log_2_ transformation (i.e. a logarithm of the number with base 2) can be applied to further stabilize variance and reduce skewness. Scaling by unit variance is available to standardize features so that each contributes equally to the analysis regardless of original scale. As an open-source software, Rodin allows users to implement specific imputation, normalization, and scaling methods of their choice. After downloading the package, users can access and modify the source code to tailor the analysis to their needs. After feature preprocessing, the data is structured into a matrix with samples as rows and features as columns, with fully duplicative rows removed.

#### 2.2.3 Statistical analysis

For univariate analysis, users can use the student’s *t* test or one-way analysis of variance (ANOVA) to assess differences between two or more study groups. The two-way ANOVA evaluates the effects of two independent variables and their interaction on a dependent variable. Logistic regression and linear regression models allow users to explore relationships between outcome variables and predictor features, with options to include covariates, interaction terms and polynomial degrees for more complex modeling. The tests provide raw *P* values and Benjamini-Hochberg (BH) adjusted *P* values to account for multiple comparisons. Additionally, fold change can be calculated to quantify expression level differences relative to a user-defined reference group.

For multivariate analysis, Partial Least Squares Discriminant Analysis (PLS-DA) is available. This supervised technique models the relationship between features and class labels, identifying variables that contribute most to class separation and providing Variable Importance in Projection (VIP) scores to highlight key discriminative features. For advanced predictive modeling, Random Forest algorithms are offered for both classification and regression tasks. These ensemble methods build multiple decision trees to improve accuracy and prevent overfitting, providing feature importance scores that indicate each variable's contribution to the model.

#### 2.2.4 Data visualization

Users can create two-dimensional (2D) plots using dimensionality reduction techniques such as Principal Component Analysis (PCA), Uniform Manifold Approximation and Projection (UMAP), and t-distributed Stochastic Neighbor Embedding (t-SNE). These methods reduce high-dimensional data into two dimensions, allowing users to identify clustering patterns, trends, and outliers within their datasets. Users can customize these plots by applying significance cutoffs to select features, identifying outliers and adjusting visual attributes like color coding (hue), marker styles, and plot titles. To visualize differential expression, volcano plots display log fold changes against the negative logarithm of *P* values with a base of 10 (−log_10_*P*) as well as VIP or importances score, and with *m*/*z* or retention time on *x*-axis and −log_10_P on *y*-axis, Manhattan plots can be generated using volcano plot function. These interactive, customizable plots are efficient in detecting features or biomarkers with both large-magnitude changes and high statistical significance. Additionally, users of the Rodin Python library can choose to export figures in higher resolution or in SVG format for publication-quality graphics.

To examine global relationships and patterns among features and samples, Rodin includes clustergrams to generate heatmaps of feature intensities with two-way unsupervised hierarchical clustering on samples and features. Options are available to standardize data before clustering and to color-code sample labels based on group variables.

The box plot function is useful to show distribution across group variables for individual metabolic features. Users can select the type of plot (box plot, violin plot, or scatter plot), select individual feature by entering the row number, or choose to show all features identified in one or all pathways, and apply trendlines using methods like Ordinary Least Squares (OLS) or Lowess to aid in interpreting data trends.

#### 2.2.5 Pathway analysis

Rodin integrates *Mummichog* (Version 2)’s pathway analysis function (Li *et al.* 2013) to identify affected metabolic pathways in untargeted metabolomics data. Informed by the Rodin Results module, users can select the use of raw *P* values or BH adjusted *P* values and set significance cutoff to achieve the optimal number of significant features, ensuring the best performance of *Mummichog*. They can also specify the ionization mode of mass spectrometry data and choose to include the log fold change to assess the directionality of metabolite differences relative to a reference group. Rodin provides annotations, *P* value, and log_2_ fold changes for each significant metabolic feature in enriched pathways. Following pathway enrichment analysis, users can utilize the boxplot function to visualize group distributions of feature levels, either individually by row number, or collectively by pathway. This enhances the ability to interpret biological implications and facilitates hypothesis generation for further investigation.

#### 2.2.6 Results download

After completing statistical analyses, users can download their results directly from the platform. This includes the preprocessed dataset merged with the outcomes of all statistical tests conducted during the session, such as raw *P* values, BH adjusted *P* values, effect sizes, and other metrics, in the format of a csv file. Rodin uses an index system for maintain features correspondence with the original user-provided dataset’s row numbers, starting from zero. This enables users to easily cross reference and plot distribution of features to examine specific features from the original dataset or patterns identified by global visualizations like volcano plots and heatmaps. This functionality sets Rodin apart from other tools by enhancing data interpretation and providing customizable and downloadable outputs for generating publication-quality graphics.

### 2.3 Case study

**Figure 2.**
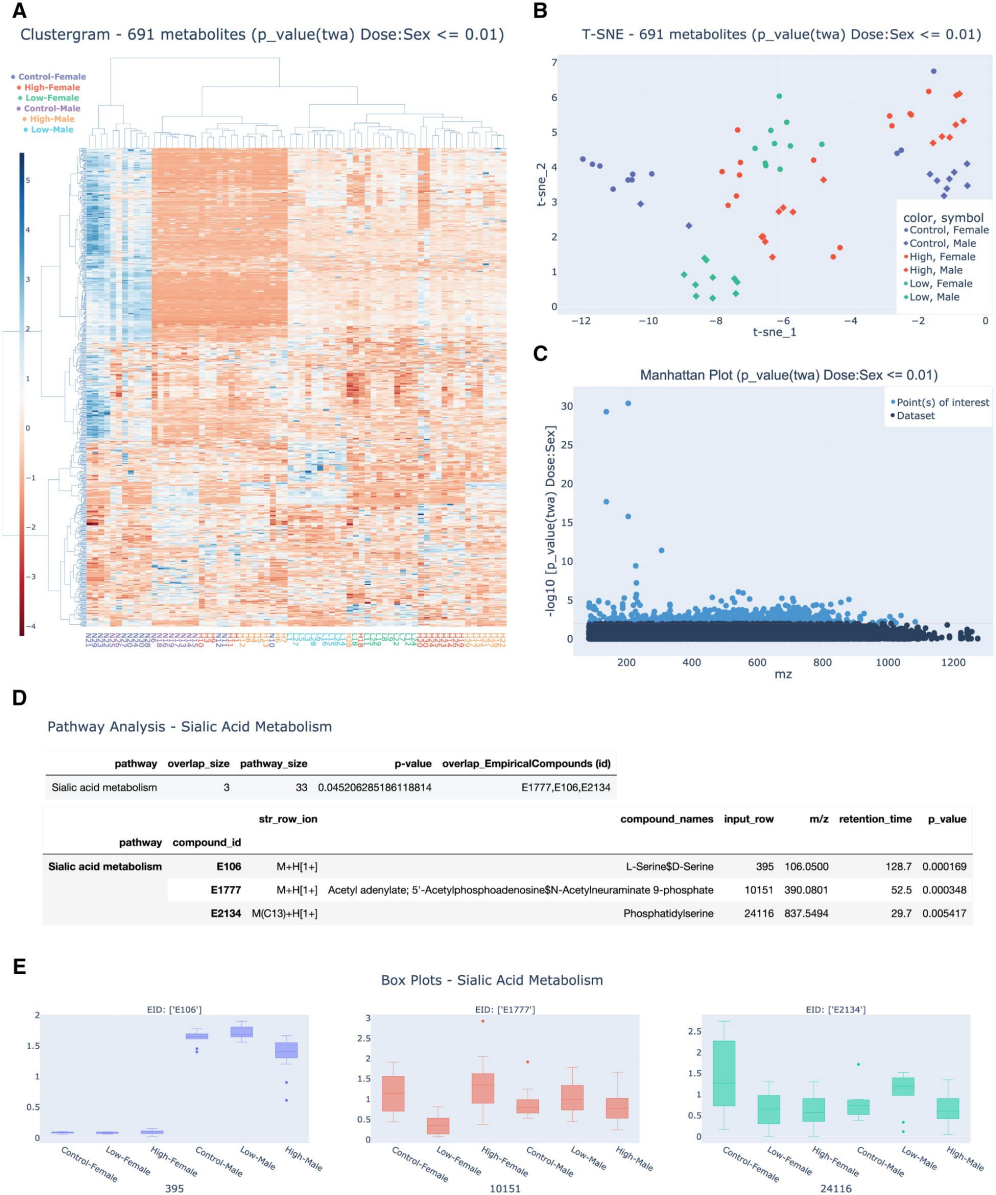
Example output in Rodin using the sample dataset collected from male and female mouse lungs that have been gestationally exposed to mixed cadmium chloride and sodium arsenite at low (50 ppb each) and high (250 ppb each) levels. (A) Clustergram of the 691 metabolites, detailing the heatmap of expression levels across different groups, with hierarchical clustering on both metabolites and sample conditions after row standardization. (B) t-SNE plot showing the clustering of 691 significant metabolites (raw *P* value < 0.01, two-way ANOVA testing interaction between dose and sex) across different exposure groups, color-coded by dose and symbol-coded by sex. (C) Manhattan plot highlighting the statistical significance (-log10 *P*) of metabolites across the mass-to-charge (m/z) spectrum, showing the distribution of *P* values to pinpoint metabolites of interest. (D) Result table of pathway analysis implementing Mummichog 2.0 showing information of metabolites in sialic acid metabolism, the pathway enriched among the 691 metabolites. (E) Box plot for selected metabolites within the Sialic Acid pathway, illustrating their distribution across the different exposure groups, separated by dose and sex.

Untargeted metabolomics data was collected from mouse lungs exposed to drinking water containing cadmium chloride and sodium arsenite at three doses—control (0 ppb), low (50 ppb), and high (250 ppb) through gestation and lactation. Lung samples were homogenized, and metabolites were extracted using established protocols of Hydrophilic Interaction Liquid Chromatography (HILIC) coupled with positive electrospray ionization (ESI). Data was extracted with apLCMS and xMSanalyzer (Yu *et al.* 2009, Uppal *et al.* 2013), generating a feature table containing 26205 features and 71 samples. The data was processed using Rodin with default settings: remove duplicates and metabolites with >50% missing values, quantile normalization, log_2_ transformation, and scaling. Two-way ANOVA was then used to test the effects of chemical dose and sex, generating global visualizations such as t-SNE and Manhattan plots on selected variables (Fig. 2A–C). The 691 features that showed a significant interaction between dose and sex with raw *P* values < 0.01 were enriched in sialic acid pathway, and the distributions of metabolites across groups were visualized (Fig. 2D and E). Currently this analysis took <2 min to complete on Rodin web interface. In contrast, using MetaboAnalyst required over 30 min, largely due to the need to manually extract the results from each analysis section, reformat the data, and re-upload it for pathway analysis.

## 3 Conclusion

Rodin is a Python-based application available via both a web interface and programmatic implementation. It streamlines metabolomics data analysis by integrating data upload, preprocessing, statistical analysis, visualization, and pathway analysis into a single, user-friendly platform. Compared to other software (Table 1), Rodin efficiently handles large datasets, addressing the need for accessible tools for scientists without programming expertise. By unifying statistical testing, feature visualization and pathway analysis, it minimizes workflow interruptions, allowing researchers to focus on result interpretation while ensuring transparency and reproducibility.

## Supplementary Material

vbaf088_Supplementary_Data

## Data Availability

The data underlying this article are available in the *rodin_guide* repository on GitHub at https://github.com/BM-Boris/rodin_guide/tree/main/data.
