# Supplementary material for: Rodin: a streamlined metabolomics data analysis and visualization tool
Source: Bioinform Adv. 2025 Apr 11;5(1):vbaf088. doi: 10.1093/bioadv/vbaf088 (PMC12034380; doi:10.1093/bioadv/vbaf088)
Supplement: vbaf088_Supplementary_Data [file vbaf088_supplementary_data.docx]

**Supplementary Materials**

**Rodin: A Streamlined Metabolomics Data Analysis and Visualization Tool**

Boris Minasenko^1^, Dongxue Wang^1^, Piera Cirillo^2^, Nickilou Krigbaum^2^, Barbara Cohn^2^, Jeffery M. Collins^3^, Dean P. Jones^4^, Xin Hu^1,4,*^

^1^ Gangarosa Department of Environmental Health, Rollins School of Public Health, Emory University, Atlanta, GA

^2^ Child Health and Development Studies, Public Health Institute, Berkeley, CA

^3^Department of Infectious Disease, School of Medicine, Emory University, Atlanta, GA

^4^ Division of Pulmonary, Allergy, Critical Care, and Sleep Medicine, Department of Medicine, School of Medicine, Emory University, Atlanta, GA

*Corresponding author ([xin.hu2@emory.edu](mailto:xin.hu2@emory.edu))

**System Compatibility**

The application has been tested across various operating systems—including Windows 10, macOS Sequoia and Monterey, and Ubuntu 20.04—as well as on multiple web browsers such as Google Chrome (version 126+), Mozilla Firefox (version 127+), Edge (version 129+) and Safari (version 16+). It has also been validated on mobile devices running iOS 16 and Android 14 with default browsers.

**Guide to Rodin (included on the Web interface)**

Data Upload:

Select and Upload Files:

Click the respective buttons to browse and select your Feature Table and Class Labels files. Ensure that both files meet the following requirements:

- Format: CSV or TXT.
- Size: Must not exceed 150 megabytes each.
- For the Feature Table:
  - For non-targeted analysis: The first two columns should include m/z values and retention times. The remaining columns should correspond to sample IDs.
  - For targeted analysis: The first column should include the annotation of metabolites.
- For the Class Labels:
  - The first column should include sample IDs, with the following columns corresponding to class labels (e.g., dose, time).
  - The sequence of sample IDs should be the same in both the Feature Table and Class Labels files.

Optional - Targeted Analysis:

If your analysis is targeted, check the "Targeted" checkbox. This setting will influence how the data is analyzed in subsequent steps.

Upload:

After selecting the files and setting the parameters, click the "Upload" button to upload the data. The system will detect the separators and prepare the data for further analysis.

Use Sample:

Alternatively, you can click the "Use Sample" button to load preloaded data for testing purposes without needing to upload your own files.

_______

 Data Preprocessing:

Select and Set Parameters:

Preprocess your data by selecting the appropriate parameters. Ensure that the following options are correctly set before proceeding:

- Threshold: Set a value between 0 and 1 to filter out features with missing values above the threshold.
- Normalization:
  - Quantile: Adjusts the distribution of data to follow a specified distribution.
  - Total Intensity: Normalizes the data by scaling each feature to a common total intensity.
  - None: No normalization will be applied.
- Log Transformation: Applies a log_2_ transformation to reduce skewness and stabilize variance in the data.
- Scaling: Scales the data by dividing each feature by its unit variance.
- Drop NaN (class): Select a specific class column to remove samples with missing values in that column.

Additional Preprocessing Details:

- Missing values will be imputed by zero during preprocessing.
- Fully duplicate rows will be automatically deleted from the dataset.

Preprocess Data:

After setting the parameters, click the "Preprocess Data" button to apply the preprocessing steps to your dataset.
Note: If the "Preprocess Data" button is clicked multiple times, the preprocessing will be applied to the already preprocessed data, potentially compounding the effects. To reapply preprocessing to the original data, click the "Upload Data" button first to reload the dataset.

_______

 Statistical Analysis:

Select and run various statistical tests to analyze your data. The available tests are described in the tabs below.
Note: Statistical tests that provide p-values will also include Benjamini-Hochberg adjusted p-values to ensure robustness against multiple testing.

T-Test

Statistical test used to determine if there is a significant difference between the means of two groups.

- Column Selection: Choose the column representing the group or class you want to compare.
- Output: The test will return the p-values for each feature, along with Benjamini-Hochberg adjusted p-values, indicating the significance of the differences.

One-Way ANOVA

Statistical test is used to determine if there are significant differences between the means of three or more independent groups.

- Column Selection: Choose the column representing the group or class you want to compare.
- Output: The test will return p-values for each feature, along with Benjamini-Hochberg adjusted p-values, indicating the significance of the differences between groups.

PLS-DA (Partial Least Squares Discriminant Analysis)

Statistical test is a supervised method that models the relationship between the features and the class labels, allowing for the identification of features that contribute the most to class separation.

- Column Selection: Choose the column representing the class or category.
- Output: The analysis will provide VIP (Variable Importance in Projection) scores, indicating the importance of each feature in discriminating between classes.

Two-Way ANOVA

Evaluates the effect of two independent variables on the dependent variable and also tests for an interaction effect between the two variables.

- Column Selection: Choose the primary column and a moderator column representing the two independent variables.
- Output: The test will return p-values indicating the significance of the main effects and the interaction effect, along with Benjamini-Hochberg adjusted p-values.

SF Logistic Regression

Statistical method used for binary classification, where the outcome variable is dichotomous (e.g., presence or absence of a condition). The analysis runs a separate regression for each feature to assess its relationship with the outcome.

- Column Selection: Choose the column representing the outcome variable, and optionally, a moderator variable.
- Output: The analysis will provide p-values, showing the significance of each feature.
- Interaction: Choose whether to include interaction terms between feature and moderator.
- Degree: Set the polynomial degree.

SF Linear Regression

Is used to model the relationship between a continuous outcome variable and each predictor feature (metabolite). The analysis runs a separate regression for each feature to assess its relationship with the outcome.

- Column Selection: Choose the column representing the continuous outcome variable, and optionally, a moderator variable.
- Output: The analysis will provide p-values for each feature, indicating the significance of the relationships.
- Interaction: Choose whether to include interaction terms between feature and moderator.

Degree: Set the polynomial degree.

Random Forest Classifier

Ensemble learning method used for classification tasks. It builds multiple decision trees and merges them together to improve accuracy and prevent overfitting.

- Column Selection: Choose the column representing the class labels.
- Output: The analysis will provide feature importance scores, indicating how much each feature contributes to the classification.
- Parameters: Set the number of trees (estimators).

Random Forest Regressor

Ensemble learning method used for regression tasks, predicting continuous outcomes by averaging the predictions of multiple decision trees.

- Column Selection: Choose the column representing the continuous outcome variable.
- Output: The analysis will provide feature importance scores.
- Parameters: Set the number of trees (estimators).

Log Fold Change

Method used to measure the change in expression levels between two conditions or groups. Note: To ensure accurate results, apply log scaling to your data before running the Log Fold Change analysis.

- Column Selection: Choose the column representing the class labels and select a reference group for comparison.
- Output: The analysis will provide log2 fold change values, indicating the magnitude of change between the groups. This output will help identify features with significant expression changes between the compared conditions.

_______

 Results

After performing the analyses, the results section displays the summarized outcomes of your statistical tests.

Download Results

Once you have reviewed the results, you have the option to download the data for further exploration or reporting. The downloaded file includes:

- The preprocessed dataset merged with the results of all statistical tests performed on the current data.
- Detailed information for each feature, including test outcomes, significance levels, and any additional metrics computed during the analysis.

Click the respective download buttons to export the results in CSV format for further use.

Note: The index of features in the results file corresponds to their position in the original data, starting from 0 for the first feature.

_______

 Data Visualization:

Use the following visualization tools to explore and present your data. The available options and their parameters are described in the tabs below.

2D Plot

2D Plots are used to reduce the dimensionality of large datasets and visualize them in two-dimensional space. The following parameters allow customization of these plots:

- Dimensionality Reduction: Choose between PCA, UMAP, or t-SNE for dimensionality reduction.
- Feature Column: Select the feature column to be used for slicing the data.
- Cutoff: Set a cutoff value to filter features based on significance or importance.
- Hue: Choose a variable to color the data points.
- Size: Select a variable to determine the size of the data points.
- Markers: Choose the marker style for the data points.
- Title: Set a custom title for the plot.

Volcano Plot

Volcano plots are used to visualize differential expression data, plotting log fold changes against -log_10_(p-value) to highlight significant features. The following parameters allow customization:

- Feature Column: Select the feature column representing p-values.
- Effect Size: Choose the column representing the effect size (e.g., log fold change).
- Significance Line: Set a threshold for p-values to identify significant features.
- Effect Size Line: Optionally, set thresholds for the effect size to highlight certain features.
- Title: Set a custom title for the plot.

Clustergram

Clustergram provides a heatmap with hierarchical clustering of both rows and columns, useful for identifying patterns and relationships between features and samples. The following parameters allow customization:

- Feature Column: Select the feature column for clustering.
- Cutoff: Set a cutoff value to filter features based on significance or importance.
- Standardize: Choose whether to standardize rows or columns before clustering.
- Hue: Choose a variable to color columns labels.
- Title: Set a custom title for the heatmap.

Box Plot

Box plots are used to visualize the distribution of data and identify outliers. The following parameters allow customization:

- Type: Choose between Box Plot, Violin Plot, or Scatter Plot.
- Rows: Specify rows to include in the plot (e.g., 2, 35, 356).
- Hue: Choose a variable to color the data points.
- Pathways: Select pathways to be included in the plot. (Note: Available when relevant data is provided.)
- Trend: Choose a trendline method such as OLS, Lowess, or Expanding.
- Title: Set a custom title for the plot.

_______

 Pathway Analysis

Pathway analysis allows to identify significantly affected pathways based on your metabolomics untargeted data.

- Feature Column: Select the column that contains the p-values. This column will be used to assess pathway enrichment.
- Mode: Choose between "Positive" or "Negative" mass spectrometry analysis mode.
- Statistics Column (optional): If you have run a Log Fold Change select the corresponding column here. This will be used in the compounds table.
- P-Value Cutoff: Set a threshold for the p-value. Only features with p-values below this cutoff will be considered significant.
- Pathways Threshold: Set a threshold for filtering pathways based on their p-values. This will help you focus on the most relevant pathways.

After setting the parameters, click the "Run Pathway Analysis" button to perform the analysis. The results will show significant pathways.

Show Compounds

After identifying significant pathways, you can explore the specific compounds involved in each pathway.

- Pathways Selection: You can choose to display compounds for all pathways or select specific pathways of interest.

Once the parameters are set, click the "Show Compounds" button to view the specific compounds involved in the selected pathways. The results will include details of the compounds, their roles in the pathways, and their significance.

Contacts

For any questions, support, or collaboration inquiries, please contact us at:

- Email: boris.minasenko@emory.edu
- Address: Rollins School of Public Health, Emory University, 1518 Clifton Road NE, Atlanta, GA 30322

You can also reach out through our GitHub repository for issues, feature requests, or contributing to the project: <https://github.com/BM-Boris/rodin>.
